# A new class of neurodegenerative disease‐fighting drugs: morADC (Morphomer®‐ antibody drug conjugates)

**DOI:** 10.1002/alz.091477

**Published:** 2025-01-09

**Authors:** Sreenivasachary Nampally, Oskar Adolfsson, Camille Martin, Romain Ollier, Sebastien Menant, Elpida Tsika, Alexis Fenyi, Nadine Ait‐Bouziad, Lorene Aeschbach, Sylvain Pautet, Aline Fuchs, David Ribas, Madiha Derouazi, Andrea Pfeifer, Marie Kosco‐Vilbois

**Affiliations:** ^1^ AC Immune SA, Lausanne, Vaud Switzerland

## Abstract

**Background:**

Antibody‐drug conjugates (ADCs) represent a major advancement in oncology to deliver selectively cytotoxic drug to tumor cells while reducing their exposure to normal tissues. Each ADC consists of a monoclonal antibody (mAb) selective to a tumor specific/overexpressed surface antigen conjugated to the cytotoxic agent. In this study, we are investigating the potential of an ADC approach in neurodegenerative diseases (NDD) to increase the exposure of therapeutic mAbs in the brain using small molecules known to be brain penetrant.

**Method:**

Several ADCs were engineered by conjugating mAbs and small molecules (SME) derived from our Morphomer® platforms, resulting in molecules called morADC. The properties of those morADCs incorporating each different mAbs, morphomers and various linkers were assessed in an *in vitro* blood‐brain‐barrier (BBB) permeability, a primary neuron seeding and Thioflavin T (ThT) aggregation assays. In the latter, the aggregates were also analyzed by electron microscopy. The most promising molecules were injected in mice to monitor brain exposure and pharmacokinetic compared to the parental mAb.

**Result:**

Over 30 morADCs were produced to target pathological proteins, e.g., Abeta, Tau and alpha‐synuclein. Drug‐to‐antibody ratios (DAR) from 2.5 to 4.5 were achieved exhibiting excellent purity and plasma stability. The morADC molecules demonstrated *in vitro* a synergistic effect quantified by isobologram analysis with 2 log higher potency in various seeding assays. When assessed *in vitro* for brain permeability, morADCs showed 3‐6‐fold enhanced BBB penetration as compared to parental mAb, dependent on the conjugated morphomer and the linker. Increased brain exposure of the morADC compared to the parental mAb was confirmed in mice: 3‐fold increase *in vitro* in the BBB permeability assay translated in 2.5‐fold increase of the mAb exposure in the mice parenchyma. The initial pharmacokinetic results indicate similar plasma concentration and stability of the morADC as the parental mAb.

**Conclusion:**

Bioconjugating functionally active mAbs and SMEs provides therapeutic morADCs as a new treatment modality with synergistic potential to target NDD pathology. More work is ongoing to better characterize the unexpected synergistic effect observed as well as proof‐of‐concept studies in relevant model pathological models. Nevertheless, our morADC platform represents a promising breakthrough approach in NDDs.

